# The Long Non-Coding RNA ENST00000489676 Influences Papillary Thyroid Cancer Cell Proliferation and Invasion through Regulating MiR-922

**DOI:** 10.7150/jca.31751

**Published:** 2019-08-29

**Authors:** Wenhan Wang, Shujun Xia, Weiwei Zhan

**Affiliations:** Department of Ultrasound, Rui Jin Hospital, Shanghai Jiao Tong University School of Medicine, Shanghai, China

**Keywords:** long noncoding RNA, ENST00000489676, papillary thyroid cancer, cell proliferation, migration, invasion, miR-922

## Abstract

**Purpose**: Long noncoding RNAs (lncRNAs) have been reported to be associated with the growth and progression of numerous cancers. The aim of this study was to explore the potential function and molecular mechanism of lncRNA ENST00000489676 in the proliferation and invasion of papillary thyroid cancer (PTC).

**Methods**: QRT-PCR (quantitative reverse transcriptase-polymerase chain reaction) was used to determine the levels of ENST00000489676. CCK-8 (Cell Counting Kit-8) assay and colony formation assay were performed to detect the cell proliferation. Flow cytometry was used to analyze the cell cycle. Transwell and scratch assay were performed to detect the migration and invasion ability.

**Results**: The expression of ENST00000489676 was significantly overexpressed in TPC1 compared to KTC1 cell lines. When ENST00000489676 expression was knocked down, the proliferation, migration and invasion ability as well as cell cycle were all promoted in PTC cell lines, while those abilities were all suppressed when ENST00000489676 overexpressed. Overexpression of ENST00000489676 could inhibit cell proliferation, migration and invasion in vitro. Moreover, ENST00000489676 may mediate tumor suppression in PTC cells through suppressing miR-922.

**Conclusions**: ENST00000489676 negatively regulated the proliferation, migration invasion and cell cycle of PTC. The overexpression of ENST00000489676 inhibited the progression of PTC through suppressing miR-922. ENST00000489676 could be act as a novel diagnosis marker and a potential therapeutic target for PTC.

## Introduction

The incidence of thyroid cancer continues to rise worldwide, which makes it the most frequent malignancies of the endocrine system 1-2. In the USA, thyroid cancer is the fifth most common cancer in women, and an estimated over 62000 new cases occurred in men and women in 2015 2. Papillary thyroid cancer (PTC), named for their papillary histological structure, is the most common subtype, accounting for more than 80% of all types of thyroid cancers 3. Although most PTC carries a good prognosis, cervical lymph nodes metastases and distant metastases such as the lungs, can cause aggressive features and poor prognosis 2. The inadequacy of treatments and lack of specific diagnostic markers are the main causes of death for PTC patients. Therefore, further researches are needed to find out more potential molecular targets and mechanisms for PTC treatments.

Long non-coding RNAs (lncRNAs) are defined as transcripts longer than 200 nucleotides without evident protein coding function 4-5. Evidence has shown that lncRNAs have biological functions, including cell growth, differentiation, progression and apoptosis 6-7. Furthermore, lncRNAs play an important role in tumor proliferation and metastasis, as many lncRNAs are dysregulated in human cancers 4. For instance, HOTAIR was proved overexpressed in breast, colon, pancreas and liver cancers 7. In human breast cancer, increased HOTAIR expression in primary tumors is correlated with metastasis and poor outcome 8. High HOTAIR expression is an independent prognostic factor for recurrence and shorter survival for the HCC patients who received a liver transplantation 9. In addition, MALAT-1 is overexpressed in different types of tumors, such as lung cancer, pancreatic cancer, breast cancer and colorectal cancer, and can influence the prognosis of tumor patients by promoting tumor growth, invasion and metastasis 10. The elevated MALAT1 expression is significantly associated with the poor overall survival 10. Besides, researches have proved that lncRNAs are closely associated with PTC pathogenesis 11-20.

To evaluate the role of lncRNAs in PTC would be of great help to understand the occurrence and progression of PTC. ENST00000489676 is one of the transcripts of TFF3, which is a member of the trefoil family and plays an important role in the formation and progressing of some cancer types 21-23.In the present study, we aimed to explore the expression level and the role of ENST00000489676 in the pathogenesis of PTC.

## Materials and Methods

### Cell culture

Human PTC cell lines, TPC1 and KTC1, were used for gain/loss-of-function study. The TPC1 was kind gifts from Key Laboratory for Endocrine and Metabolic Diseases of Chinese Health Ministry (Shanghai, China). KTC1 was purchased from Shanghai Institutes for Biological Sciences (Shanghai, China). TPC1 and KTC1 was cultured in RPMI 1640 Medium HEPES (Gibco) supplemented with 10% of FBS (Gibco, Carlsbad, CA, USA) and 100 U/ml penicillin, 100 μg/ml streptomycin. HUVEC (human umbilical vein endothelial cells) was cultured in DMEM-H (Gibco, Carlsbad, CA, USA) supplemented with 10% of FBS (Gibco) and 100 U/ml penicillin, 100 μg/ml streptomycin. Cell lines were incubated at 37℃ in a 5% CO_2_ humidified atmosphere.

### Total RNA extraction and qRT-PCR (quantitative reverse transcriptase-polymerase chain reaction)

Total RNA was extracted from the cultured cell lines using trizol reagent (Invitrogen, Carlsbad, CA, USA) according to the manufacturer's instruction. Total RNA(one microgram) was reversely transcribed into first-strand cDNA by using Reverse Transcription Kit (Takara, Dalian, China) and the subsequent PCR was carried out by using SYBR® Premix Ex TaqTM II Kit (Takara, Dalian, China) according to the instruction in VIIA7 system (Applied Biosystems, California, USA). The primer sequences of ENST00000489676 and GAPDH were showed in Table 1. All of the data were analyzed using the comparison Ct (2-^△△Ct^) method and value for ENST00000489676 expression was normalized to its GAPDH, which was applied as endogenous control in all amplifications. Each sample test was analyzed in triplicate independently.

### Cell lines transfection

For loss-of-function study, RNA interference was used to knock down the expression of ENST00000489676 in KTC1 cell line. ENST00000489676 specific shRNA1, shRNA2 and scramble shRNA (negative control) were purchased from GenePharma Co., Ltd (Shanghai, China). The sense and antisense sequences were showed in Table 1. ENST00000489676 specific shRNA1, shRNA2 and scramble shRNA were constructed into pLKO vector and then transfected into KTC1 cell using LipofectamineTM2000 (Thermo Fisher Scientific, Waltham, MA, USA) according to the manufacturer's instruction. The expression levels of ENST00000489676 were confirmed by qRT-PCR analysis respectively.

For gain-of-function study, lentiviral transduction was applied to overexpress the expression of ENST00000489676 in TPC1 cell line. ENST00000489676 was ligated into pLVX to construct ENST00000489676 overexpression plasmid. The pLVX empty vector (negative control, NC) and pLVX- ENST00000489676 constructs were transfected into the HEK293T viral packaging cell line. Forty eight hours after transfection, the viral supernatant was collected and used for the infection of cells. TPC1 cells were infected with viral suspension for 24h before the assay. We obtained stably transfected clones by Puromycin (Promega, 2μg/ml) selection. The TPC1 cells were infected with pLVX- ENST00000489676 and pLVX-NC by using LipofectamineTM2000 (Life Technologies) according to the manufacturer's instruction and antisense oligonucleotides were transfected at the concentration of 100nM. The expression level of ENST00000489676 was confirmed by qRT-PCR respectively.

### Scratch assay

Forty eight hours after transfection, cells were plated in each well of the 6-well plate and incubated to form 100% confluence. Subsequently, a scratched was performed with pipette tips. Fresh serum-free medium was replaced. The wound closing procedure was observed for 24h, and images were taken. All experiments were performed independently in triplicate.

### Transwell assay

Transwell chambers (Costar, Corning, NY, USA) were used to perform migration assay and invasion assay. For transwell migration assay, the upper chamber was added with 200ul serum-free medium while the lower chamber was placed with 600ul medium with 5% FBS. The number of 1×10^4^ cells was placed into the upper chamber. For transwell invasion assay, matrigel (BD Biosciences, New Jersey, USA) was coated on the top side of the insert membrane before the medium was added. The chambers were maintained in 37℃ and 5% CO_2_ humidified atmosphere for 24h. Then the un-migrated or un-invaded cells on the top side of the insert membrane were removed with cotton swabs. The insert membranes were then fixed in methanol for 20 minutes and stained with 1% crystal violet for 30 minutes. The migrated or invaded cells on the bottom of the membrane were calculated under microscopy and images were captured. All experiments were performed independently in triplicate.

### Colony formation assay

Forty-eight hours after transfection, TPC1 and KTC1 cells were seeded in 6-well plates with a density of 1×10^3^cells/well and cultured at 37℃ in a 5% CO_2_ humidified atmosphere. The cultured medium was replaced every the other day. After 7 days' culture, the medium was removed and cells were washed twice with PBS. Cells were stained with crystal violet for 30 minutes at room temperature, then washed again and images were photographed.

### CCK-8 analysis

Forty-eight hours after transfection, TPC1 and KTC1 cells were seeded into the 96-well plate with a density of 1×10^4^ cells/well. Cell viability was assessed by cell counting kit-8 (CCK-8; Dojindo Molecular Technologies, Japan) at 24h, 48h, 72h and 96h. The absorbance was determined by TECAN infinite M200 plate reader at 450nm.

### Flow cytometry assay

Forty-eight hours after transfection, TPC1 and KTC1 cells were collected for cell cycle and apoptosis assay. For cell cycle analysis, cells were fixed in 75% ethanol overnight at 4 ℃. Cell Cycle and Apoptosis Analysis Kit (Beyotime biotechnology, Jiangsu, China) was applied to stain the treated cells according to the manufacturer's instruction. Then, the cells were analyzed by Gallios Flow Cytometry (Beckman Coulter, USA) in order to quantify the proportion of cells in each stage of cell cycle (S, G1, G2/M). For apoptosis assay, the Alexa Fluor® 488 Annexin V/Dead Cell Apoptosis Kit (Life technology) was used. The treated cells were suspended by 100 μl binding buffer with 5 μl Annexin V and 1μl propidium iodide, followed by 15 minutes' incubation protected from light. Then 400 μl binding buffer was added and the cells were resuspended. Gallios Flow Cytometry (Beckman Coulter, USA) was applied to quantify the proportion of cells marked with Annexin V and propidium iodide. All experiments were performed independently in triplicate.

### Statistical analysis

All tests and calculations were performed with GraphPad Prism 5.0 (GraphPad Software, LaJolla, CA, USA) and Statistical Program for Social Sciences 19.0 software (SPSS, Chicago, IL, USA). Independent two-sample t test was used to compare the expression level of ENST00000489676 in the cell lines. P<0.05 was considered to indicate a statistically significant difference.

## Results

### The expression of ENST00000489676 in PTC cell

**Fig 1:**
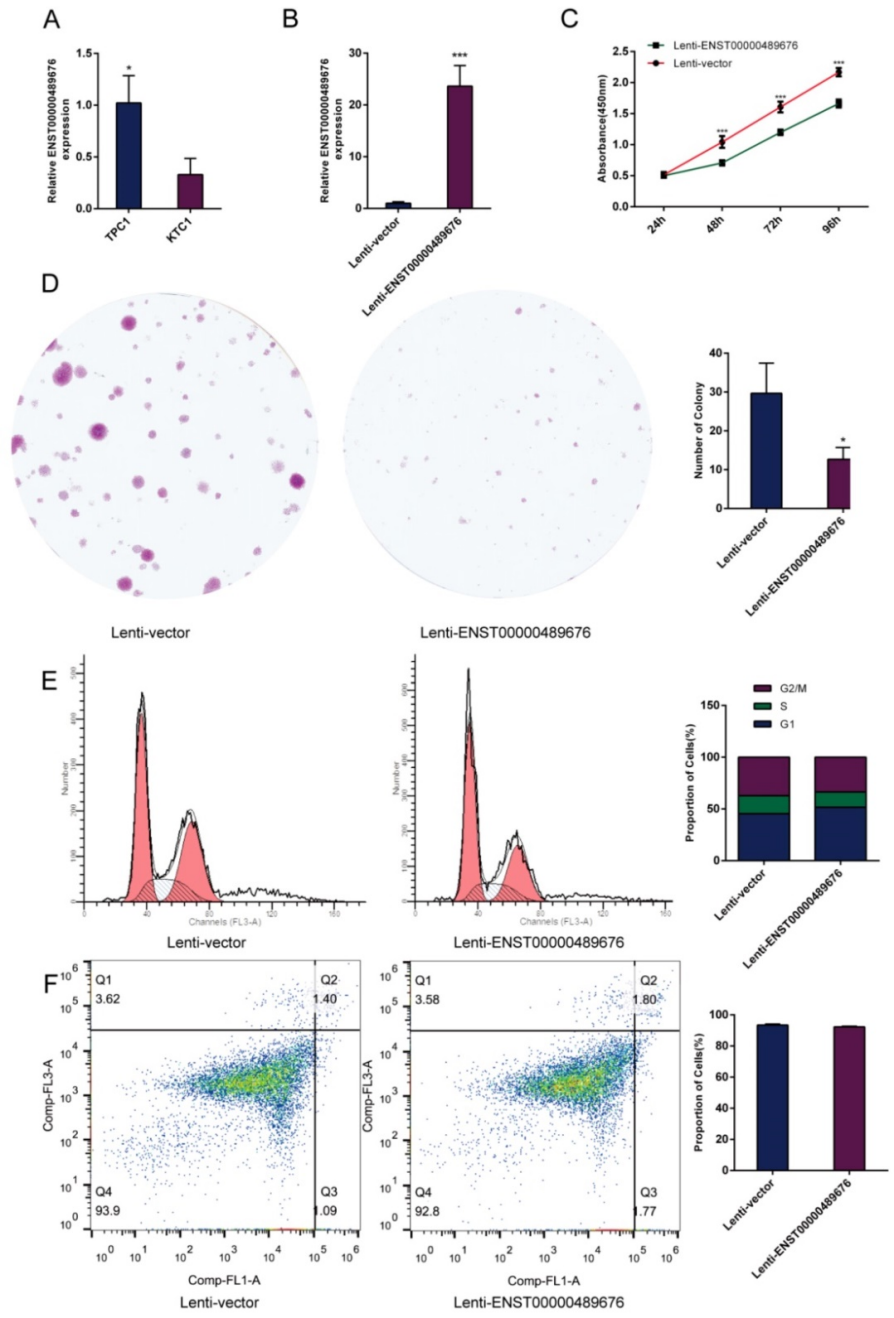
** The impact of ENST00000489676 overexpression on proliferation, cell cycle and apoptotic ability of KTC1 cells**.(A) qRT-PCR analysis of the expression level of ENST00000489676 in KTC1 and TPC1 cells. (B) qRT-PCR analysis of the expression level of ENST00000489676 in KTC1 cells infected with Lenti-vector and Lenti-ENST00000489676. The values were normalized to GAPDH mRNA expression. (C) CCK-8 assay was performed to determine the KTC1 cell proliferation ability after 24hours, 48hours, 72 hours and 96 hours with ENST00000489676 overexpressed. (D)Colony formation assay was performed to determine the proliferation ability of KTC1 cells infected with Lenti-vector and Lenti-ENST00000489676. The colonies were captured and counted. The number of colonies was presented in histogram. (E) Flow cytometry images of cell cycle in KTC1 cells infected with Lenti-vector and Lenti-ENST00000489676. Results quantified in cell cycle were showed as a percentage of total cells. (F) Flow cytometry images of cell apoptosis in KTC1 cells infected with Lenti-vector and Lenti-ENST00000489676. Results quantified in viable cells were showed as a percentage of total cells. All data were expressed as mean ±SD of three independent experiments (“*” indicates P<0.05, “***” indicates P<0.001).

**Fig 2:**
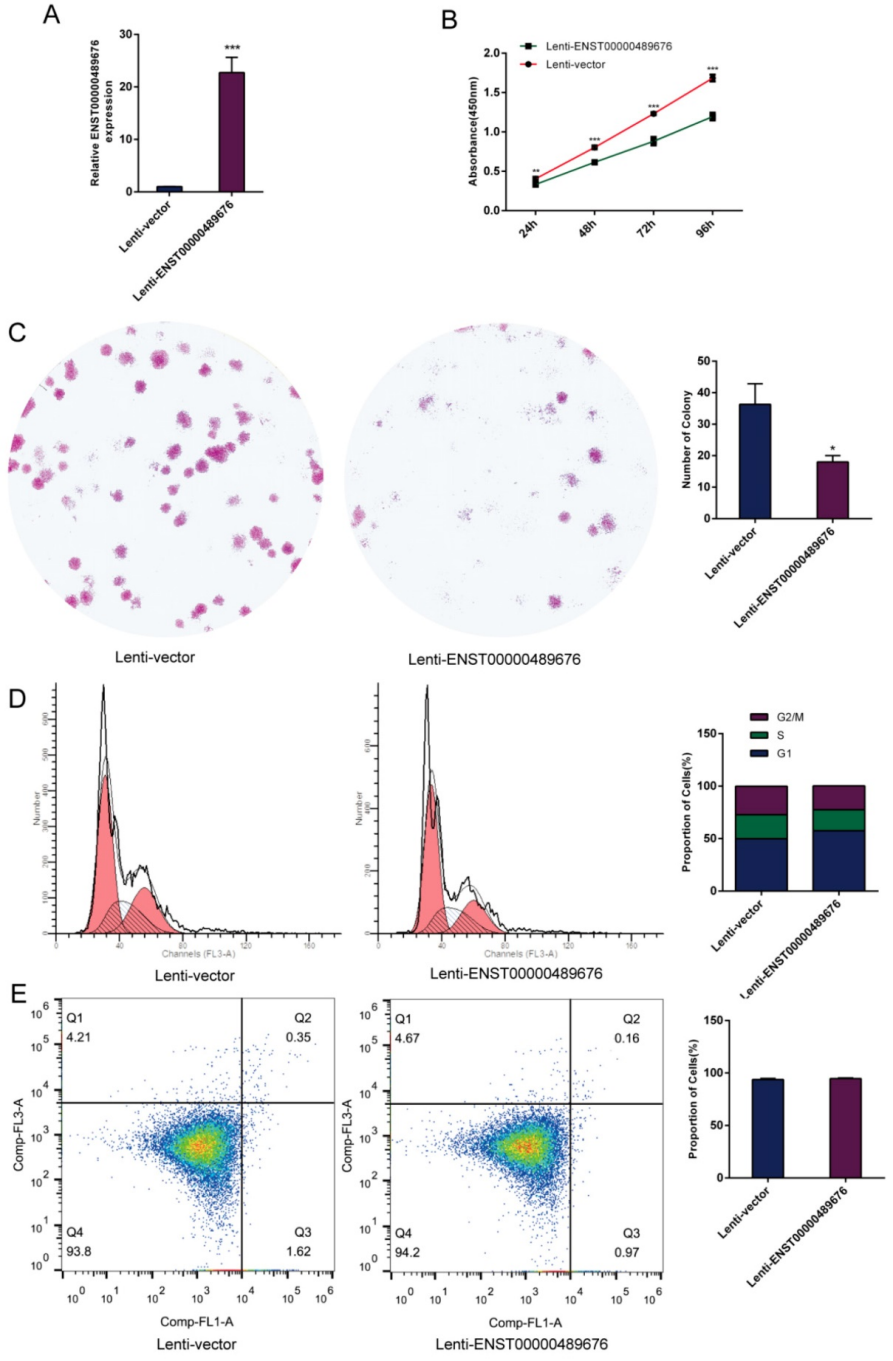
** The impact of ENST00000489676 overexpression on proliferation, cell cycle and apoptotic ability of TPC1 cell.**(A) qRT-PCR analysis of the expression level of ENST00000489676 in TPC1 cells infected with Lenti-vector and Lenti-ENST00000489676. The values were normalized to GAPDH mRNA expression. (B) CCK-8 assay was performed to determine the TPC1 cell proliferation ability after 24hours, 48hours, 72 hours and 96 hours with ENST00000489676 overexpressed. (C)Colony formation assay was performed to determine the proliferation ability of TPC1 cells infected with Lenti-vector and Lenti-ENST00000489676. The colonies were captured and counted. The number of colonies was presented in histogram. (D) Flow cytometry images of cell cycle in TPC1 cells infected with Lenti-vector and Lenti-ENST00000489676. Results quantified in cell cycle were showed as a percentage of total cells. (E) Flow cytometry images of cell apoptosis in TPC1 cells infected with Lenti-vector and Lenti-ENST00000489676. Results quantified in viable cells were showed as a percentage of total cells. All data were expressed as mean ±SD of three independent experiments (“*” indicates P<0.05, “**” indicates P<0.01, “***” indicates P<0.001).

**Fig 3:**
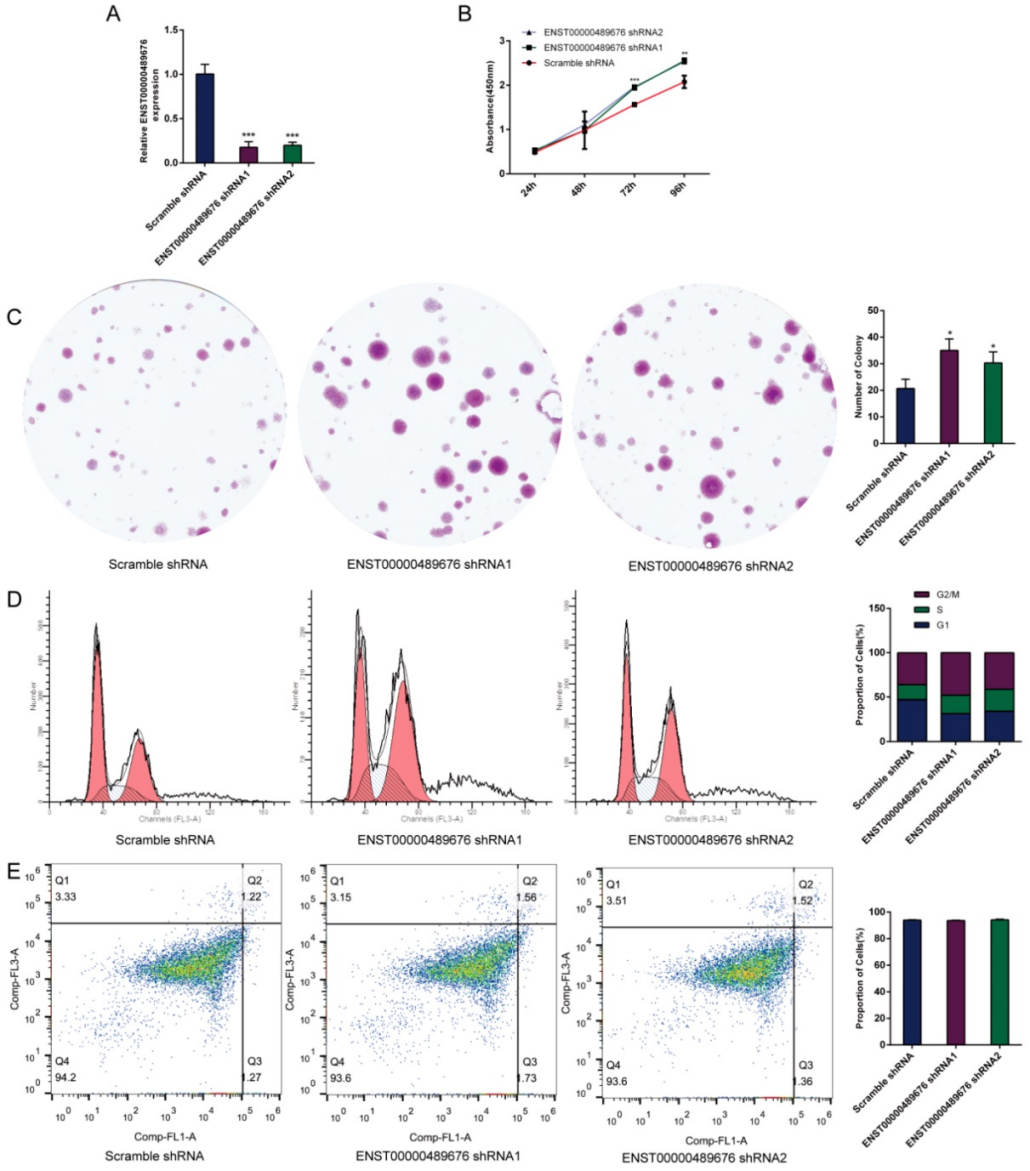
** The impact of ENST00000489676 knockdown on proliferation, cell cycle and apoptotic ability of KTC1 cell.**(A) qRT-PCR analysis of the expression level of ENST00000489676 in KTC1 cells infected with pLKO-NC, pLKO-shRNA1 and pLKO-shRNA2. The values were normalized to GAPDH mRNA expression. (B) CCK-8 assay was performed to determine the KTC1 cell proliferation ability after 24hours, 48hours, 72 hours and 96 hours with ENST00000489676 knocked down. (C)Colony formation assay was performed to determine the proliferation ability of KTC1 cells infected with pLKO-NC, pLKO-shRNA1 and pLKO-shRNA2. The colonies were captured and counted. The number of colonies was presented in histogram. (D) Flow cytometry images of cell cycle in KTC1 cells infected with pLKO-NC, pLKO-shRNA1 and pLKO-shRNA2. Results quantified in cell cycle were showed as a percentage of total cells. (E) Flow cytometry images of cell apoptosis in KTC1 cells infected with pLKO-NC, pLKO-shRNA1 and pLKO-shRNA2. Results quantified in viable cells were showed as a percentage of total cells. All data were expressed as mean ±SD of three independent experiments (“*” indicates P<0.05, “**” indicates P<0.01, “***” indicates P<0.001).

**Fig 4:**
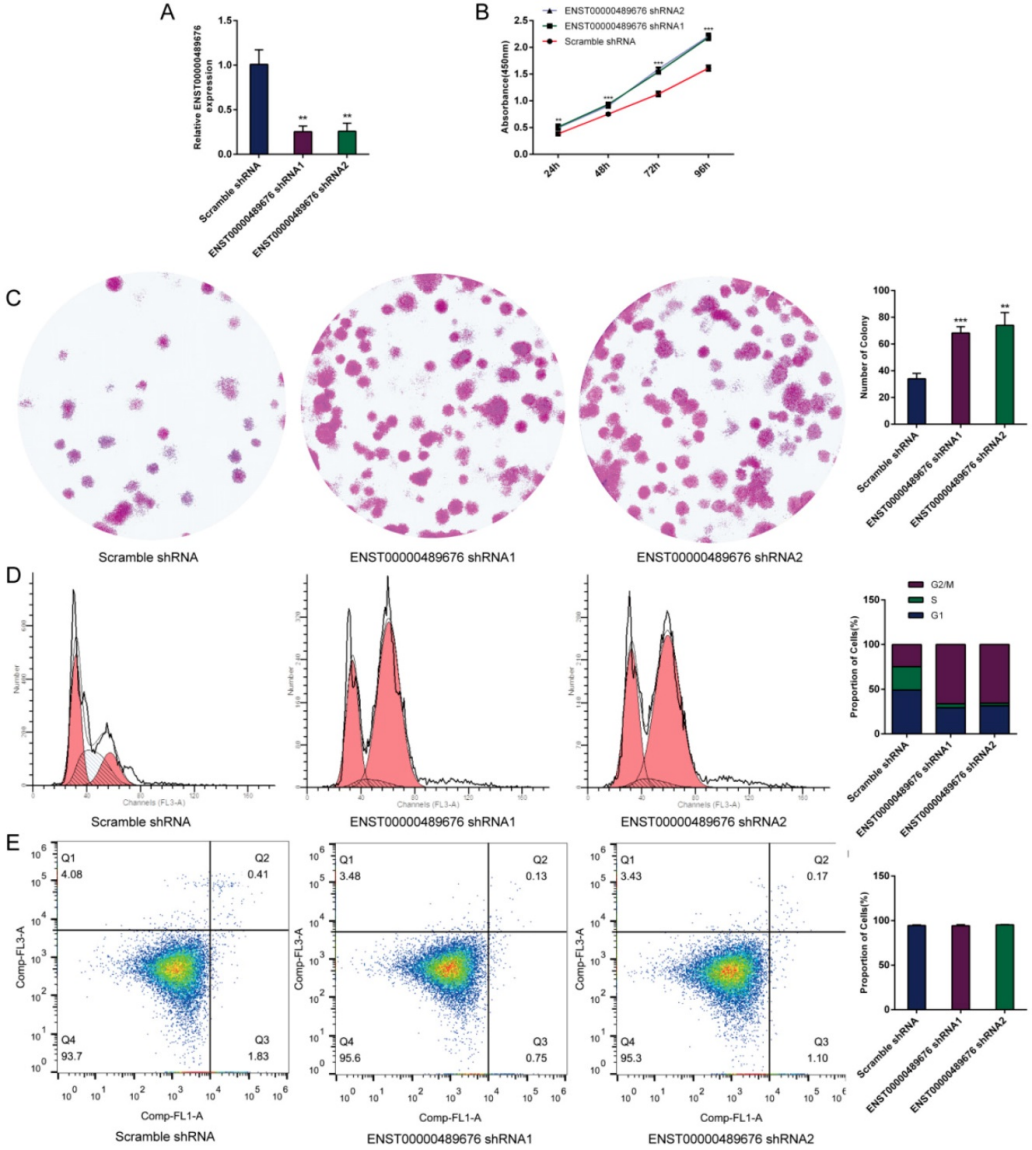
** The impact of ENST00000489676 knockdown on proliferation, cell cycle and apoptotic ability of TPC1 cell.**(A) qRT-PCR analysis of the expression level of ENST00000489676 in TPC1 cells infected with pLKO-NC, pLKO-shRNA1 and pLKO-shRNA2. The values were normalized to GAPDH mRNA expression. (B) CCK-8 assay was performed to determine the TPC1 cell proliferation ability after 24hours, 48hours, 72 hours and 96 hours with ENST00000489676 knocked down. (C)Colony formation assay was performed to determine the proliferation ability of TPC1 cells infected with pLKO-NC, pLKO-shRNA1 and pLKO-shRNA2. The colonies were captured and counted. The number of colonies was presented in histogram. (D) Flow cytometry images of cell cycle in TPC1 cells infected with pLKO-NC, pLKO-shRNA1 and pLKO-shRNA2. Results quantified in cell cycle were showed as a percentage of total cells. (E) Flow cytometry images of cell apoptosis in TPC1 cells infected with pLKO-NC, pLKO-shRNA1 and pLKO-shRNA2. Results quantified in viable cells were showed as a percentage of total cells. All data were expressed as mean±SD of three independent experiments (“**” indicates P<0.01, “***” indicates P<0.001).

The expression level of ENST00000489676 was analyzed in KTC1 and TPC1 cell lines. The qRT-PCR analysis showed that ENST00000489676 expression was significantly lower in KTC1 cell line with comparison to that in TPC1 cell line (Fig. 1A). For loss-of-function study, both KTC1 and TPC1 cells were transfected with pLKO-NC (Scramble shRNA), pLKO-shRNA1 (ENST00000489676 shRNA1), and pLKO-shRNA2 (ENST00000489676 shRNA2). For gain-of-function study, both KTC1 and TPC1 cells were transfected with lenti-vector and lenti-ENST00000489676. The transfection efficiency was confirmed by qRT-PCR analysis. The expression level of ENST00000489676 was significantly overexpressed in KTC1 (Fig. 1B) and TPC1 cells (Fig. 2A) after transfection with lenti-ENST00000489676, while it was knocked down in KTC1 (Fig. 3A) and TPC1 cells (Fig. 4A) after transfection with pLKO-shRNA1 and pLKO-shRNA2.

### ENST00000489676 regulates the proliferation ability of PTC cell

CCK8 assay and colony formation assay were performed to detect the proliferation ability of KTC1 and TPC1 cells. In CCK8 assay, the proliferation ability of KTC1 and TPC1 cell were examined at four time points (24h, 48h, 72h, 96h) after transfection. The cell viability was showed as proliferation curve based on the absorbance at 450nm. In Lenti-ENST00000489676 group, the cell viabilities of KTC1 and TPC1 were both suppressed compared to the Lenti-vector group (Fig. 1C, 2B). In pLKO-shRNA1 and pLKO-shRNA2 group, the cell viabilities of KTC1 and TPC1 were both promoted compared to the pLKO-NC group (Fig. 3B, 4B). In colony formation assay, the number of colonies in Lenti-ENST00000489676 group was less than that in Lenti-vector group (Fig. 1D, 2C), while there were more number of colonies in pLKO-shRNA1 and pLKO-shRNA2 group compared to the pLKO-NC group (Fig. 3C, 4C). These results indicated that ENST00000489676 negatively regulated the proliferation of PTC.

### ENST00000489676 regulates the cell cycle and apoptosis of PTC cell

The flow cytometry was used to analyze the cell cycle of KTC1 and TPC1 cells after transfection. It showed that the proportions of cells at G1 phase was significantly higher in Lenti-ENST00000489676 group compared to that in Lenti-vector group, and the proportions of cells was less at G2/M, S phase in Lenti-ENST00000489676 group compared to control (Fig. 1E, 2D). Conversely, the proportions of cells at G1 phase was significantly lower in pLKO-shRNA1 and pLKO-shRNA2 group compared to that in pLKO-NC group, while that was more at G2/M, S phase in pLKO-shRNA1 and pLKO-shRNA2 group compared to the control (Fig. 3D, 4D). Likewise, the apoptotic ability of KTC1 and TPC1 cells after transfection was examined by flow cytometry assay, which showed that there was no significant difference as regard to the proportion of apoptotic cells between Lenti-vector and Lenti-ENST00000489676 group (Fig. 1F, 2E); and the difference of apoptotic cells among pLKO-NC, pLKO-shRNA1 and pLKO-shRNA2 group was recognized as well (Fig. 3E, 4E). These results indicate that the cell cycle of PTC was significantly impacted by dysregulation of ENST00000489676, while the cell apoptotic ability was not influenced by ENST00000489676 expression.

### The impact of ENST00000489676 on the migration and invasion ability of PTC cell

**Fig 5:**
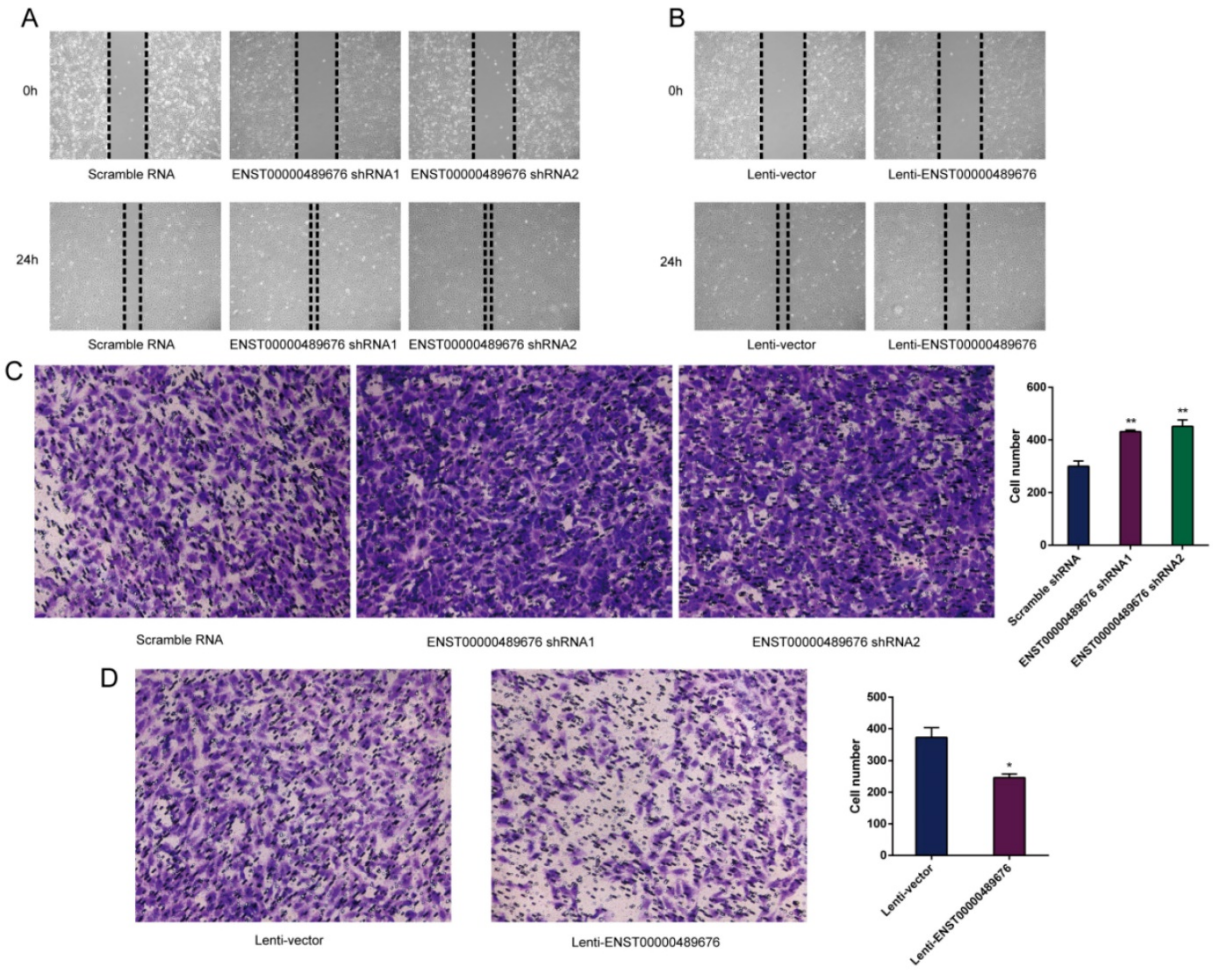
** The impact of ENST00000489676 knockdown and overexpression on migration and invasion of KTC1 cell.** Wound healing assay was performed to determine the migration ability of KTC1 cells with ENST00000489676 knocked down (A) and overexpressed (B). Representative images of 0 hour and 24 hours of three repeated experiments were showed. Transwell assay was performed to determine the invasion ability of KTC1 cells with ENST00000489676 knocked down (C) and overexpressed (D). The representative images of invaded cells at lower chamber were stained with crystal violet. The quantifications of cell invasion were presented as invaded cell numbers. All data were expressed as mean±SD of three independent experiments (“*” indicates P<0.05, “**” indicates P<0.01).

**Fig 6:**
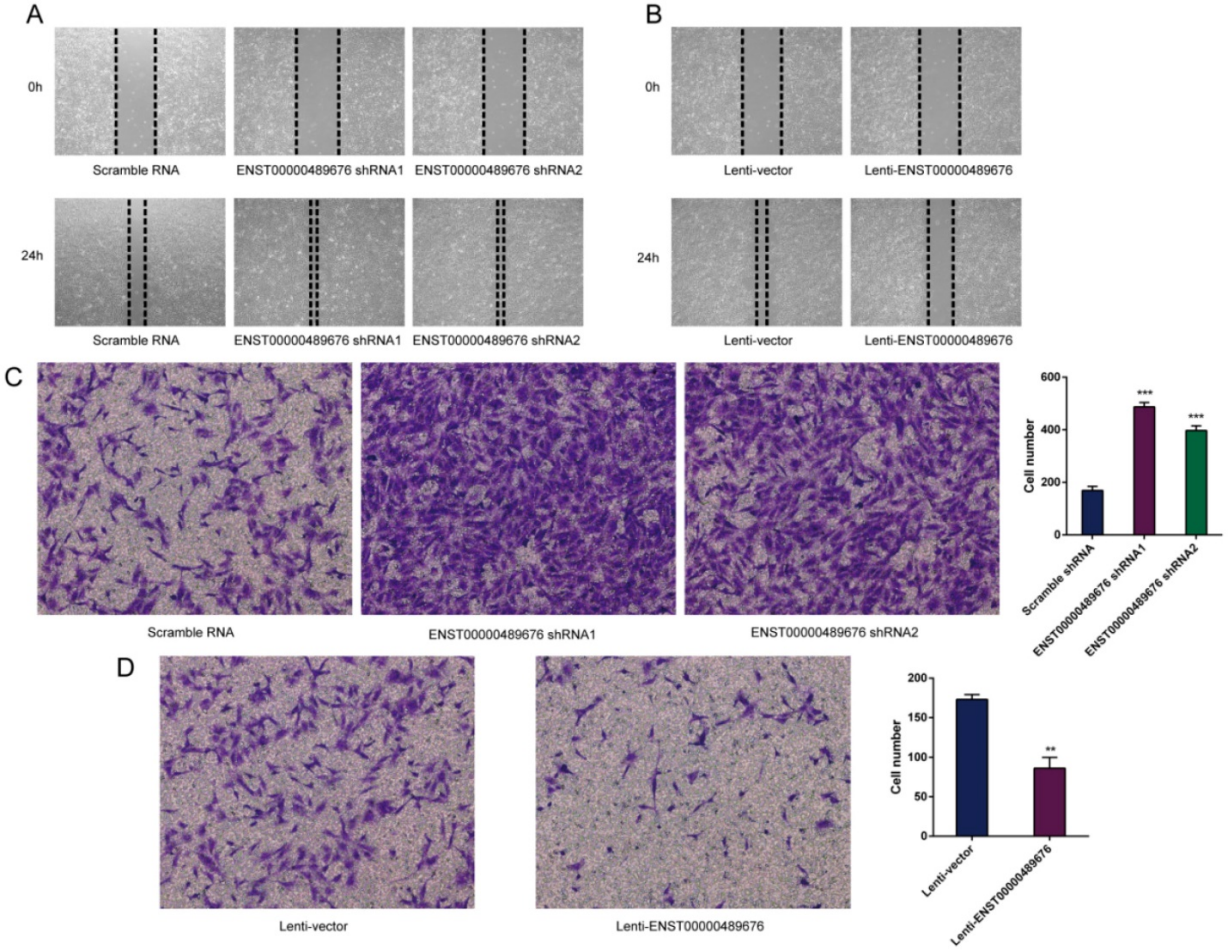
** The impact of ENST00000489676 knockdown and overexpression on migration and invasion of TPC1 cell.** Wound healing assay was performed to determine the migration ability of TPC1 cells with ENST00000489676 knocked down (A) and overexpressed (B). Representative images of 0 hour and 24 hours of three repeated experiments were showed. Transwell assay was performed to determine the invasion ability of TPC1 cells with ENST00000489676 knocked down (C) and overexpressed (D). The representative images of invaded cells at lower chamber were stained with crystal violet. The quantifications of cell invasion were presented as invaded cell numbers. All data were expressed as mean±SD of three independent experiments (“**” indicates P<0.01, “***” indicates P<0.001).

The scratch assay was performed to study the migration ability of KTC1 and TPC1 cell after transfection. The distance of the scratch wound in Lenti-ENST00000489676 group was significantly larger compared to the Lenti-vector group (Fig. 5B, 6B), while it was significantly smaller in pLKO-shRNA1 and pLKO-shRNA2 group with comparison to pLKO-NC group (Fig. 5A, 6A). In addition, transwell invasion assay was used to investigate the invasive ability of KTC1 and TPC1 cell after transfection. The number of cells invaded through the chamber in Lenti-ENST00000489676 group was significantly less than that in the Lenti-vector group (Fig. 5D, 6D). Conversely, the number of cells invaded through the chamber in pLKO-shRNA1 and pLKO-shRNA2 group was significantly more than that in the pLKO-NC group (Fig. 5C, 6C). These results indicated that ENST00000489676 negatively regulated the migration and invasion of PTC.

### Tumor cell-derived ENST00000489676 regulates endothelial cell migration and invasion

**Fig 7:**
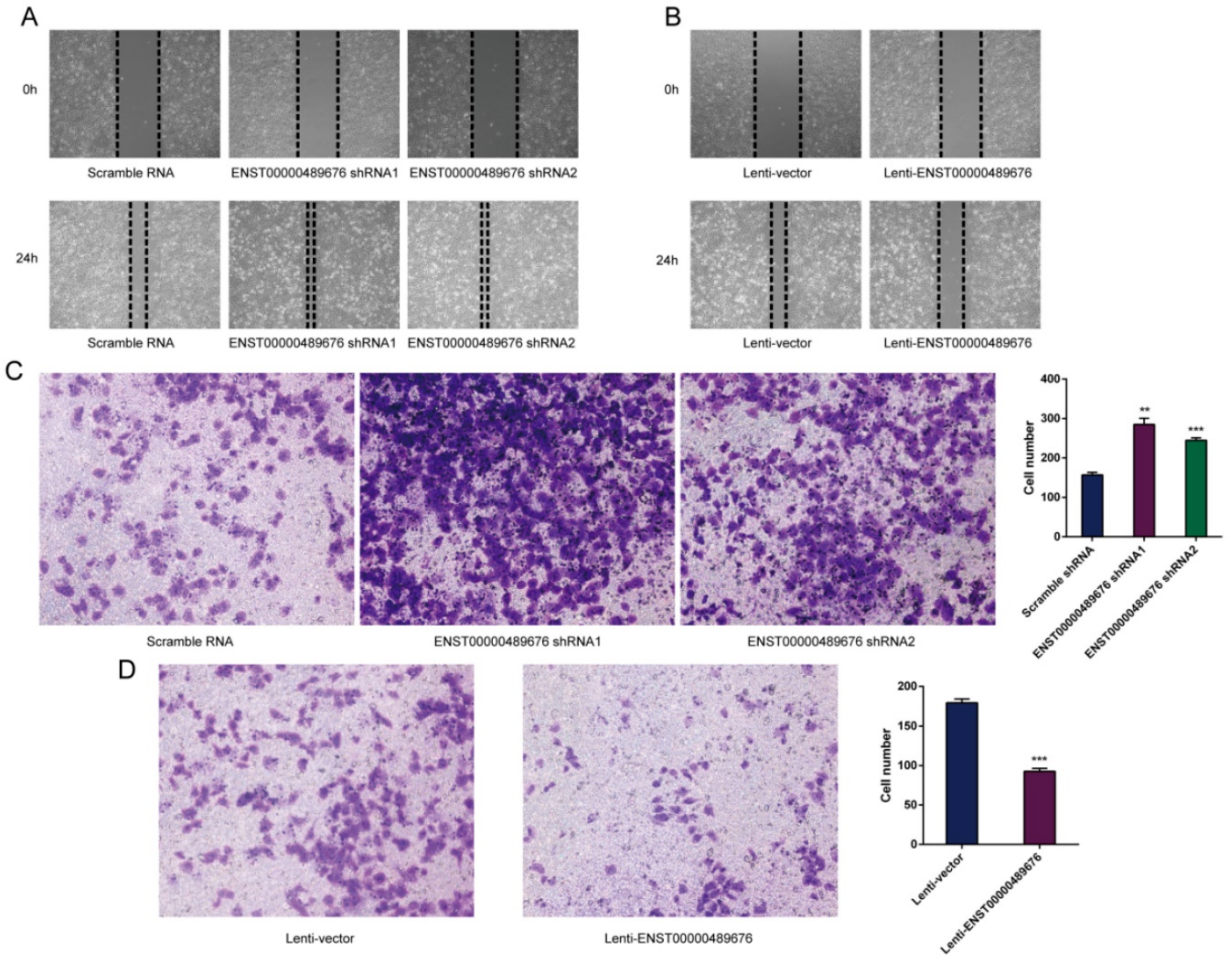
ENST00000489676 knockdown and overexpression regulate the impact of KTC1 cell on endothelial cell migration and invasion. HUVECs were incubated with the supernatant of KTC1 cells transfected with pLKO-NC, pLKO-shRNA1, pLKO-shRNA2, Lenti-vector and Lenti-ENST00000489676 respectively. Then HUVECs were subjected to wound healing assay and matrigel invasion assay. ENST00000489676 knockdown and overexpression in KTC1 cells affect the migration (A) (B) and invasion (C) (D) ability of endothelial cells. Representative micrographs of HUVECs stimulated with supernatants of ENST00000489676 knocked down and overexpressed KTC1 cells were shown. All data were expressed as mean±SD of three independent experiments (“**” indicates P<0.01, “***” indicates P<0.001).

**Fig 8:**
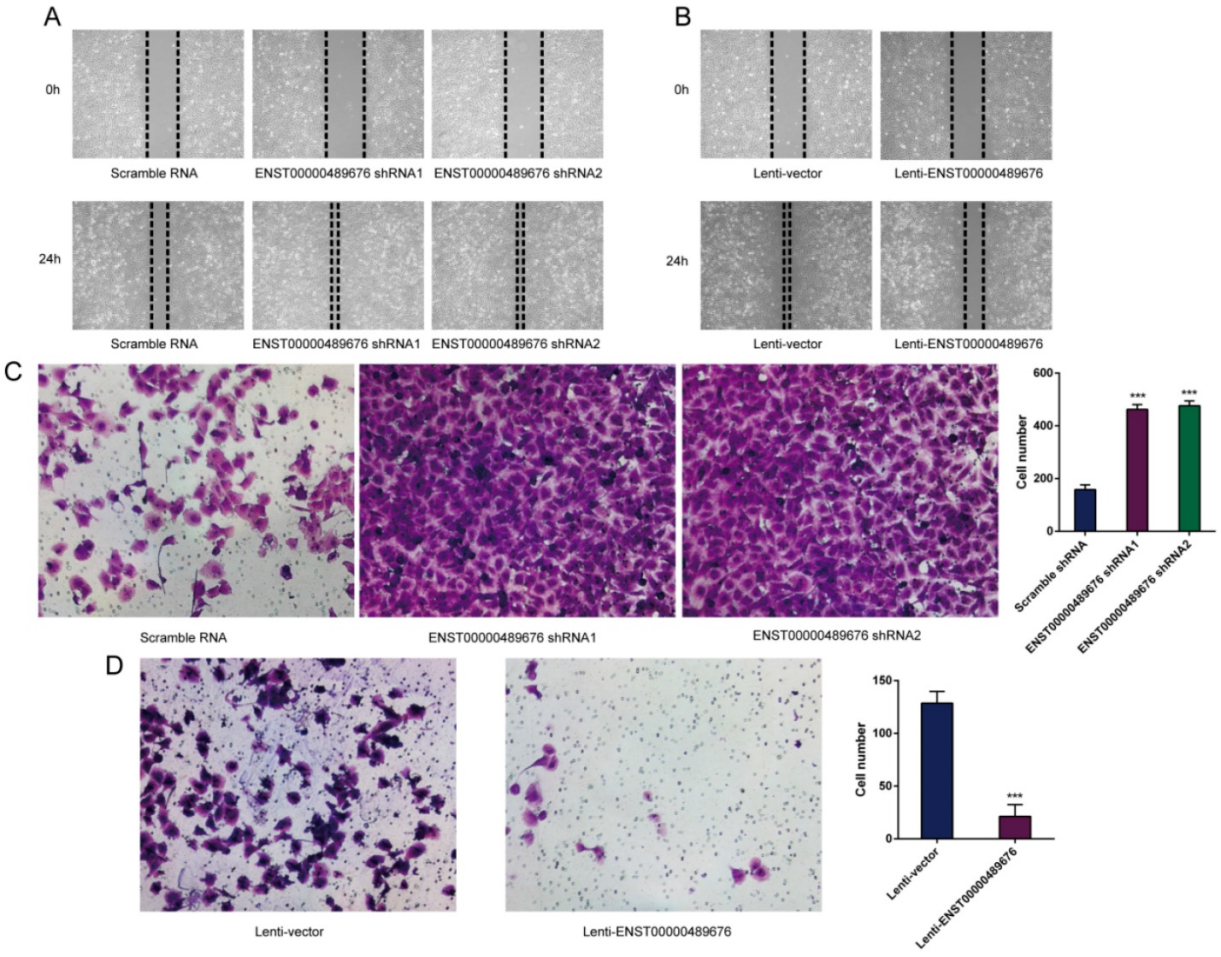
** ENST00000489676 knockdown and overexpression regulate the impact of TPC1 cell on endothelial cell migration and invasion.** HUVECs were incubated with the supernatant of TPC1 cells transfected with pLKO-NC, pLKO-shRNA1, pLKO-shRNA2, Lenti-vector and Lenti-ENST00000489676 respectively. Then HUVECs were subjected to wound healing assay and matrigel invasion assay. ENST00000489676 knockdown and overexpression in TPC1 cells affect the migration (A) (B) and invasion (C) (D) ability of endothelial cells. Representative micrographs of HUVECs stimulated with supernatants of ENST00000489676 knocked down and overexpressed TPC1 cells were shown. All data were expressed as mean±SD of three independent experiments. ( “***” indicates P<0.001).

This study further explored the migration and invasion function of KTC1 or TPC1 cell-derived ENST00000489676 on the surrounding endothelial cells. HUVECs were incubated with conditioned media from ENST00000489676 overexpressed or knocked down KTC1 or TPC1 cell. The wound healing assay was performed to detect the migration ability of HUVECs, showing that the distance of the scratch wound in Lenti-ENST00000489676 group was significantly larger compared to the Lenti-vector group (Fig. 7B, 8B), while it was significantly smaller in pLKO-shRNA1 and pLKO-shRNA2 group with comparison to pLKO-NC group (Fig. 7A, 8A). Moreover, the transwell invasion assay was performed to detect the invasion ability of HUVECs after incubated with supernatant from ENST00000489676 overexpressed or knocked down KTC1 or TPC1 cell. The data showed that the number of cells invaded through the chamber in Lenti- ENST00000489676 group was significantly less than that in Lenti-vector group (Fig. 7D, 8D), while it was significantly more in pLKO-shRNA1 and pLKO-shRNA2 group compared to the pLKO-NC group (Fig. 7C, 8C).These results indirectly indicated that ENST00000489676 negatively regulated the migration and invasion of PTC.

### Overexpression of ENST00000489676 inhibits PTC cell invasion by suppressing miR-922 expression

**Fig 9:**
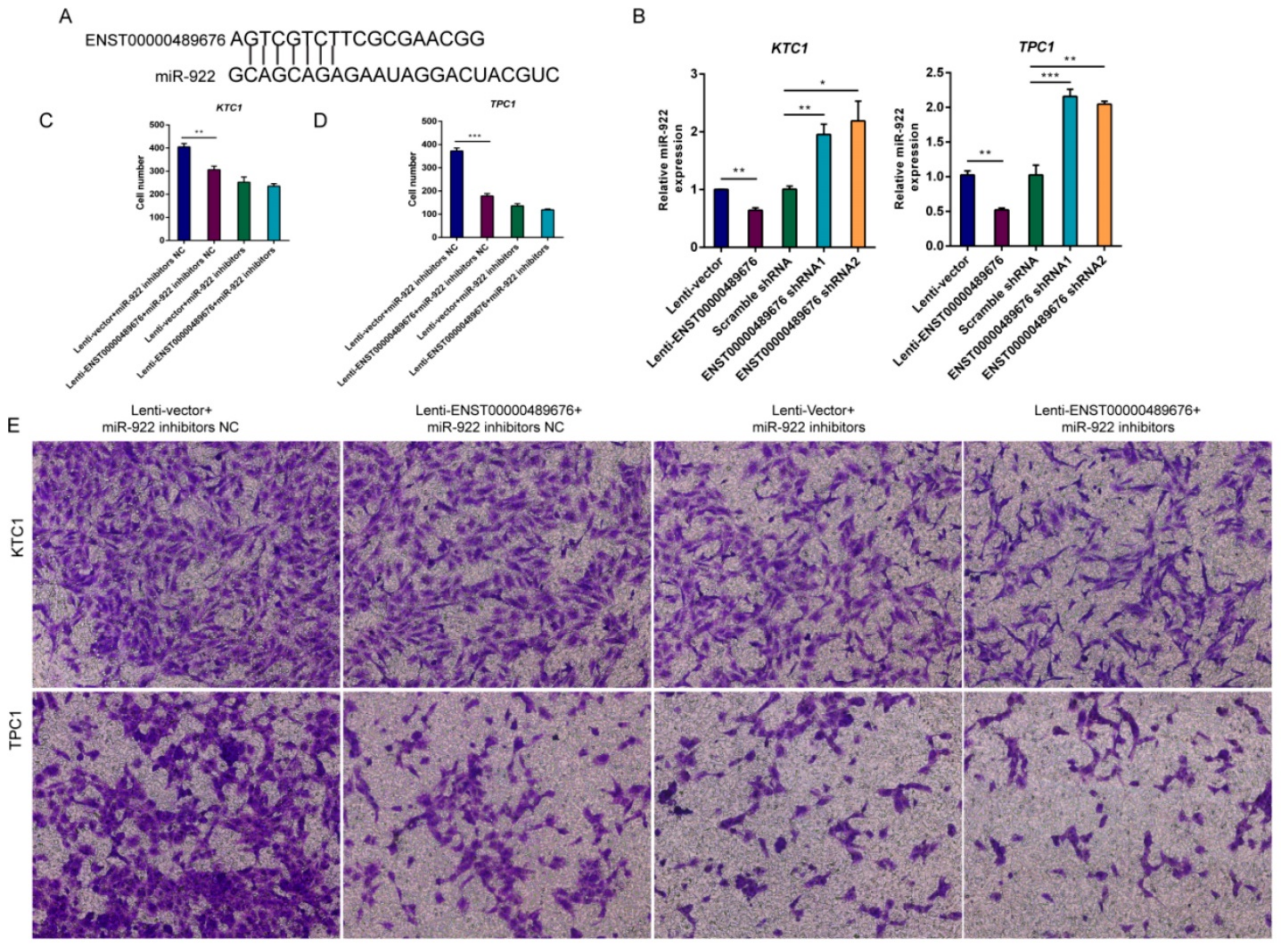
** ENST00000489676 regulates KTC1 and TPC1 cell invasion by negatively modulating miR-922.** (A)The bioinformatic analysis under MIRDB4.0(http://mirdb.org/miRDB/index.html) predicted a target site on ENST00000489676 that was complementary with miR-922;(B) qRT-PCR analysis of the expression level of miR-922 in KTC1 or TPC1 cells infected with Lenti-vector, Lenti-ENST00000489676, pLKO-NC, pLKO-shRNA1 and pLKO-shRNA2 respectively. (C) (E) Transwell assay was performed to determine the invasion ability of KTC1 cells under co-transfection of Lenti-ENST00000489676 and miR-922 inhibitors. (D) (E) Transwell assay was performed to determine the invasion ability of TPC1 cells under co-transfection of Lenti-ENST00000489676 and miR-922 inhibitors. The representative images of invaded cells at lower chamber were stained with crystal violet. The quantifications of cell invasion were presented as invaded cell numbers. All data were expressed as mean±SD of three independent experiments ( “*” indicates P<0.05 ,“**” indicates P<0.01, “***” indicates P<0.001).

Through MIRDB4.0 software for bioinformatic analysis (http://mirdb.org/miRDB/index.html), there was a target site on ENST00000489676 that was complementary with miR-922 (Fig. 9A), indicating that ENST00000489676 may interact with miR-922 in PTC. Then, we performed the qRT-PCR to detect the expression of miR-922 in KTC1 and TPC1 after ENST00000489676 was overexpressed or knocked down respectively. The data showed that miR-922 expression was significantly lower in Lenti-ENST00000489676 group than in Lenti-vector group while it was significantly higher in ENST00000489676 shRNA1 and shRNA2 group than in Scramble RNA group both in KTC1 and TPC1 (Fig. 9B). Thus, dysregulated ENST00000489676 impacted miR-922 expression in PTC. Furthermore, transwell invasion assay was done in order to confirm whether ENST00000489676 influenced PTC invasion through regulating the function of miR-922. Using miR-922 inhibitors to suppress the function of miR-922, we found that there was no significant difference between Lenti-ENST00000489676 group and Lenti-vector group as regard to the invasion ability of KTC1 and TPC1 (Fig. 9D, 9E). However, when using miR-922 inhibitors NC as a control, there was a decreased invasion ability of KTC1 and TPC1 in Lenti-ENST00000489676 group compared to that in Lenti-vector group (Fig. 9C, 9E). These data indicated that ENST00000489676 regulated PTC cell invasion by negatively modulating miR-922 expression.

## Discussion

The continuing rising incidence of PTC has made it a public health problem worldwide 2. Although most PTC carries a good prognosis after surgical excision combined with radioiodine and levothyroxine treatment, metastasis and recurrence occur in some PTC patients, which leads to a poor prognosis 2. The investigation of the role of dysregulated molecules in PTC would help us understand the mechanism of pathogenesis and progression, and also find out the effective treatment strategies for aggressive PTC.

Currently, various lncRNAs have been demonstrated to be associated with biological functions including invasion and progression of PTC (Table 2). These studies indicated that lncRNAs may act as potential targets for PTC diagnosis and treatment. For instance, Xia et al proved that overexpressed lncRNA CCND2-AS1 promoted the ability of proliferation, migration, and invasion in PTC 12. Li et al reported that HIT000218960 was significantly upregulated in PTC and the increased HIT000218960 expression level was associated with metastatic phenotype such as lymph node metastasis 13. Qin et al demonstrated for the first time that the overexpression of GAS8-AS1 inhibited proliferation and significantly increased activation of autophagy in PTC cell lines 14. Zhou et al showed that lncRNA CASC2 expression was significantly downregulated in PTC tissues and the overexpression of lncRNA CASC2 inhibited cell proliferation, migration and invasion of PTC 15.

In the current study, lncRNA ENST00000489676 was reported in the field of thyroid cancer for the first time. ENST00000489676, with a length of 419 nucleotides, was discovered as one of the non-protein-coding transcripts of TFF3, which has been reported to be involved in the biological activities of tumors. Elnagdy et al studied TFF3 in the blood of breast cancer patients and found that TFF3 was upregulated in the blood of metastatic breast cancer 24. Taniguchi et al reported the association of TFF3 expression with gastric cancer, demonstrating that TFF3 overexpression was significantly correlated with the depth of invasion, lymph node metastasis and shorter recurrence-free survival 25. We assumed that ENST00000489676 might also be involved in regulating carcinogenesis or tumor invasion. In the current study, the phenotype of ENST00000489676 was confirmed in vitro. In the loss of function study, when ENST00000489676 expression was knocked down, the proliferation, migration and invasion ability as well as cell cycle were all promoted in PTC cell lines, while those abilities were all suppressed in gain of function study with ENST00000489676 overexpressed. Therefore, ENST00000489676 may play a negative regulating role in PTC growth and progression.

During the process of tumor progression, different lncRNAs play critical roles through different mechanisms. CeRNA (competing endogenous RNAs) is one of the common mechanisms that lncRNAs involve. H19 is a well-known and extensively explored lncRNA in regulating tumor activities. In nasopharyngeal carcinoma, H19 promoted invasive properties via inhibition of miR-630 and the subsequent regulation of enhancer of zeste homolog 2 (EZH2), which is a target of the miRNA 26. Another study demonstrated the role of H19 in larynx squamous cell carcinoma and showed that H19 suppressed the activity of miR-148a-3p, and then enhanced the expression of the target gene DNMT1, which promoted proliferation, migration, and invasion of larynx squamous cell carcinoma cells 27. The above studies both illustrated that lncRNA H19 acted as ceRNA for miRNAs. In the current study, the influence of ENST00000489676 on PTC cell invasion was not impacted when miR-922 expression was inhibited at the same time. Thus, miR-922 was the downstream of ENST00000489676. It could be assumed that ENST00000489676 might be as ceRNA against miR-922, which deserves further investigation.

In conclusion, ENST00000489676 played an important role in regulating proliferation, migration invasion and cell cycle of PTC. The overexpression of ENST00000489676 inhibited the progression of PTC through suppressing miR-922. This provided a new experimental basis to explore the effects of lncRNA in the diagnosis and treatment of PTC. ENST00000489676 may be a predictor for invasion and metastasis of PTC such as lymph nodes metastasis, and may serve as a potential diagnostic and therapeutic target.

## Figures and Tables

**Table 1:** The primer sequences of ENST00000489676 and GAPDH.

Gene/shRNA		Sequence(5'-3')
ENST00000489676	Forward Primer	GGCAGCTGACAGGGCTTT
	Reverse Primer	GCAGTCCACCCTGTCCTTG
GAPDH	Forward Primer	AAGGTGAAGGTCGGAGTCAAC
	Reverse Primer	GGGGTCATTGATGGCAACAATA
		
ENST00000489676-shRNA1	Sense	GCAAGCGCTTCTGCTGAAAGT
	Antisense	ACTTTCAGCAGAAGCGCTTG
ENST00000489676-shRNA2	Sense	GCCTGATGTCTTAACGAATAA
	Antisense	TTATTCGTTAAGACATCAGGC
Scramble shRNA	Sense	AGCATCGTACGTAGGCCAG
	Antisense	CTGGCCTACGTACGATGCT

**Table 2:** Some recent researches about lncRNAs and the biological functions in PTC

LncRNAs	Expressions in PTC	Biological functions in PTC
CCND2-AS1^12^	upregulated	Promote progression, migration and invasion.
HIT000218960 13	upregulated	Promote oncogenesis and tumor progression.
FOXD2-AS1^16^	upregulated	Promote progression and migration
AFAP1-AS1^17^	upregulated	Promotes tumor growth and metastasis.
HOTTIP^18^	upregulated	Promoted proliferation, invasion and migration
GAS8-AS1 14	downregulated	Inhibited proliferation.
CASC2^15^	downregulated	Inhibited proliferation, migration and invasion.
BLACAT1^19^	downregulated	As a predicting marker of lymph node metastasis
LINC00312^20^	downregulated	Inhibited proliferation, migration and invasion.
